# Risk Factors and Prediction Model for Early‐Onset Immune‐Related Adverse Events in Pan‐Cancer Patients Undergoing Anti‐PD‐(L)1 Therapy: A Retrospective Study in a Tertiary‐Level Hospital

**DOI:** 10.1002/cam4.71603

**Published:** 2026-02-10

**Authors:** Panpan Jiao, Lijuan Xue, Weijuan Tan, Quan Chen, Shan Lin, Min Song, Chunling Ma, Juan Zhan

**Affiliations:** ^1^ Department of Oncology Zhongshan Hospital Affiliated to Xiamen University Xiamen Fujian China; ^2^ School of Pharmaceutical Sciences Xiamen University Xiamen Fujian China; ^3^ Department of Pharmacy Zhongshan Hospital Affiliated to Xiamen University Xiamen Fujian China

**Keywords:** immune‐related adverse events, immunotherapy, prediction model, risk factors

## Abstract

**Background:**

Anti‐programmed death 1 (PD‐1) and anti‐programmed death ligand 1 (PD‐L1) immune checkpoint inhibitors (ICIs) have changed the treatment landscape of many advanced malignancies. However, immune‐related adverse events (irAEs) bring great challenges to clinical benefits. The prediction of irAEs is urgently demanded for early detection and intervention.

**Methods:**

Patients in our center who received anti‐PD‐(L)1 immunotherapy between January 2019 and May 2023 were collected. Logistic least absolute shrinkage and selection operator (LASSO) regression analysis with 10‐fold cross‐validation was performed to identify the most relevant variables associated with irAEs. Multivariate logistic regression analysis was used to build a prediction model by introducing features selected in LASSO regression analysis.

**Results:**

Overall, 680 eligible patients were included, of whom 330 patients were included in the irAEs group. In the irAEs group, 455 different irAEs were reported, of which 52 events were grade 3 or higher in severity. Endocrinal toxicities (174/680, 25.59%) were the most commonly reported irAEs. Through LASSO and logistic regression analysis, we developed a risk assessment model to predict the risk of irAEs based on basophil percentage (BASO%), hemoglobin (Hb), absolute lymphocyte count (ALC), platelet‐to‐lymphocyte ratio (PLR), lymphocyte‐to‐monocyte ratio (LMR), blood urea nitrogen level (BUN), the Charlson comorbidity index (CCI) score, Eastern Cooperative Oncology Group Performance Status (ECOG PS), and hepatitis B/hepatitis B surface antigen carriers. The model had a *C*‐index of 0.727, with good discrimination and calibration capabilities.

**Conclusion:**

The prediction model developed in our study can screen and monitor patients with high risk of developing irAEs. It may improve prognosis for pan‐cancer patients receiving anti‐PD‐(L)1 immunotherapy.

## 1 Introduction

In the past few years, immune checkpoint inhibitors (ICIs) have profoundly changed the treatment landscape of many advanced malignancies. However, ICIs use is usually followed by a remarkable spectrum of therapy‐associated organ toxicities, called immune‐related adverse events (irAEs). Although any tissue or organ may be affected, irAEs are primarily related to the skin, endocrine glands, gastrointestinal tract, lung, and liver [1]. Most irAEs manifest as mild conditions, but a few can be life‐threatening, such as pneumonitis, neurological disorders, and myocarditis. Pneumonitis, the most common fatal irAE, results in a 28% death rate in patients receiving anti‐programmed death 1 (PD‐1) or anti‐programmed death ligand 1 (PD‐L1) antibody therapy [2, 3]. IrAEs may hinder clinical benefits and restrain ICIs use, leading to worse prognosis in cancer patients. Therefore, early prediction of irAEs is crucial, as it can help identify high‐risk patients prone to developing irAEs and intervene promptly.

Previous literature has indicated that several potential biomarkers could serve as early predictive factors for irAEs. These biomarkers were mainly obtained from peripheral blood parameters, such as neutrophil‐to‐lymphocyte ratio (NLR) [4], platelet‐to‐lymphocyte ratio (PLR) [5] and lymphocyte‐to‐monocyte ratio (LMR) [6]. Genomics [7], cytokines [8, 9], autoantibodies [10] as well as tumor microenvironment [11, 12, 13] can also serve as biomarkers, but they were not usually used in real clinical practice. Besides, several models have been developed to predict irAEs, such as the cytokine toxicity score for assessing severe immune‐related toxicities in melanoma patients and a model comprising 9 lipids for predicting irAEs in non‐small cell lung cancer (NSCLC) patients [14, 15]. However, these biomarkers and prediction models had not covered other malignancies and cannot accurately predict irAEs.

Artificial intelligence (AI) is rapidly advancing in oncology, especially as it is increasingly integrated into precision medicine and drug response prediction [16, 17]. Within the context of immunotherapy, AI‐based methods have expanded the horizon for discovering efficacy biomarkers, spanning genomic, radiomic, pathomic, and real‐world evidence [18]. Critically, AI also holds significant promise for predicting and mitigating treatment‐related adverse events [19]. Zhong et al. constructed a predictive model based on clinical data using machine learning methods, which can accurately assess the risk of early cognitive impairment in patients with hypertension [20].

In this retrospective study, we aimed to investigate patients' risk factors related to irAEs and develop a predictive model for evaluating the risk of irAEs, enabling early risk screening and intervention for pan‐cancer patients undergoing ICIs.

## 2 Methods

### 2.1 Study Design and Participants

**FIGURE 1:**
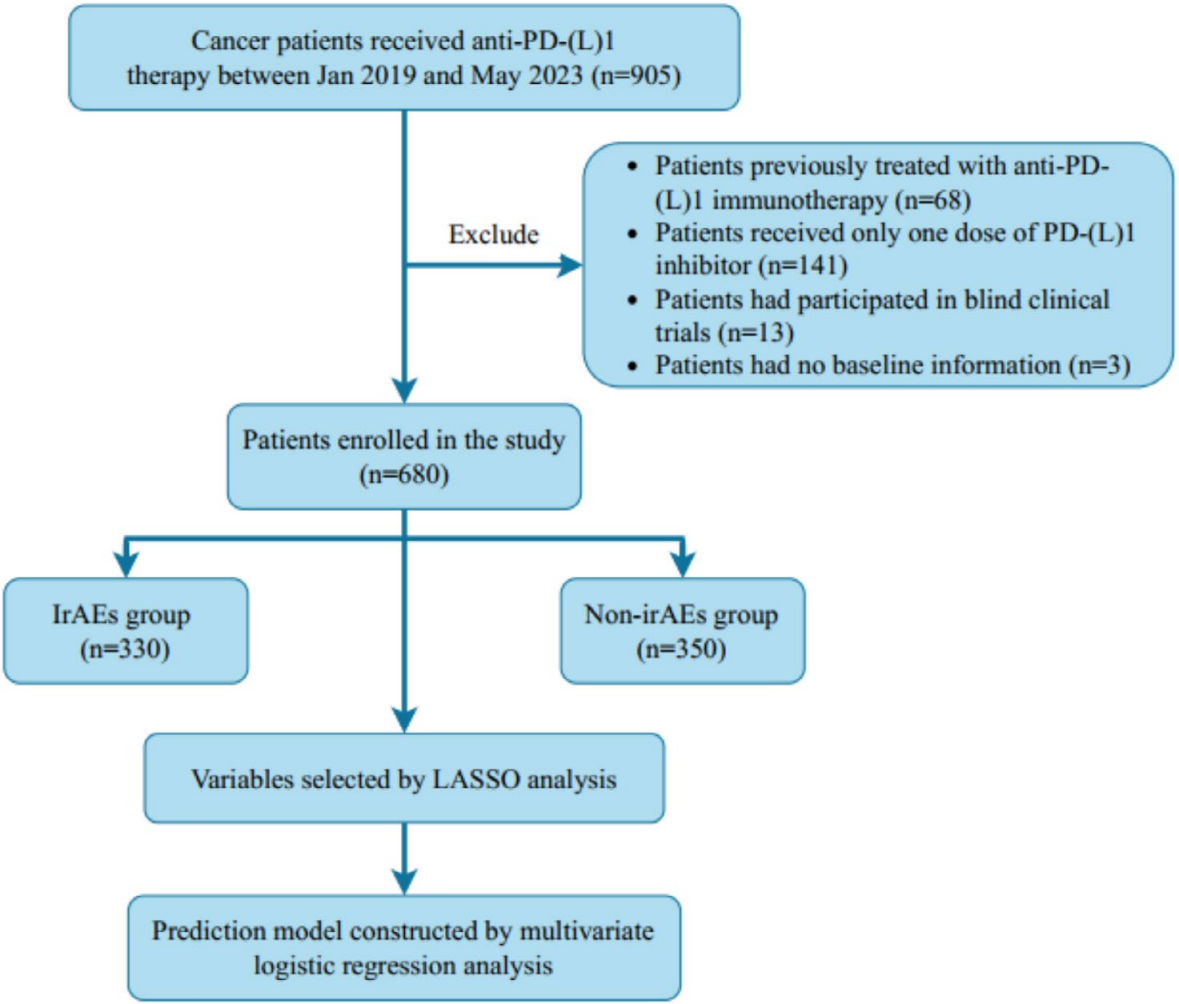
Flowchart of the study.

Patients with a confirmed pathological diagnosis of cancer who received anti‐PD‐(L)1 immunotherapy in Zhongshan Hospital affiliated to Xiamen University between January 2019 and May 2023 were screened in our study. All patients were ≥ 18 years old and their cancers were in active stage. The exclusion criteria were as follows: (i) patients previously treated with anti‐PD‐(L)1 immunotherapy; (ii) patients received only one dose of PD‐(L)1 inhibitor; (iii) patients had participated in blind clinical trials; (iv) patients had no baseline information or complete follow‐up information. Finally, a total of 680 patients were enrolled in this study. Figure 1 shows the procedure of our study.

### 2.2 Data Collection

Patient characteristics were obtained from the medical records system. We collected information about age, sex, Eastern Cooperative Oncology Group Performance Status (ECOG PS), body mass index (BMI), history of smoking, tumor type, clinical stage, comorbidities, etc. The Charlson comorbidity index (CCI) was calculated to quantitatively assess comorbidities in individual patients. Baseline blood data (defined as those taken within 1 week before ICIs initiation), including complete blood counts and biochemical indicators, were collected. NLR (absolute neutrophil count/absolute lymphocyte count), PLR (absolute platelet count/absolute lymphocyte count), LMR (absolute lymphocyte count/absolute monocyte count), derived neutrophil and lymphocyte ratio (dNLR, neutrophils/[leukocyte‐neutrophils]), prognostic nutrition index (PNI, serum albumin value, g/L + 5 × total lymphocyte count, 10^9^/L), systemic immune‐inflammatory index (SII, NLR × platelet count), and the Geriatric Nutritional Risk Index (GNRI, 1.489 × serum albumin value, g/L + 41.7 × BMI/22) were calculated.

### 2.3 Definition of irAEs

The Common Terminology Criteria for Adverse Events (CTCAE) of the National Cancer Institute (version 5.0) was used to determine patients' adverse events. The irAEs are defined as inflammatory side effects induced by ICIs, through increasing the activity of the immune system. In our study, irAEs were evaluated within 3 months after ICIs treatment initiation, because the incidence of irAEs had been reported to reach a climax within the first 12 weeks [21, 22]. Patients were included in the irAEs group if they had at least one irAE.

### 2.4 Statistical Analysis

The continuous variables were expressed by mean ± standard deviation or median (25th, 75th), and the categorical variables were expressed by number (percentage %). We performed *T*‐test or Mann–Whitney test for the continuous variables, and the categorical variables were analyzed using Chi‐square tests or Fisher exact tests. Logistic least absolute shrinkage and selection operator (LASSO) regression analysis with 10‐fold cross‐validation was performed to identify the most relevant variables associated with irAEs. Multivariate logistic regression analysis was used to build a prediction model by introducing the features selected in LASSO regression analysis. A *p* value < 0.05 was considered statistically significant.

## 3 Results

### 3.1 Patient Characteristics

Overall, 680 eligible patients were included in our study. Patient characteristics are summarized in Table 1. The median age was 61 years (range 22–87), and a preponderance of sex was male (76.62%). A majority of patients had an ECOG PS of 0/1 (91.62%). The most common tumor type was NSCLC (26.47%), followed by liver cancer (20.44%) and esophageal cancer (12.21%). Of note, 631 patients were at stage III/IV prior to ICIs treatments (92.79%). Additionally, 278 patients received ICIs monotherapy (40.88%) while 402 patients were prescribed combination therapies (59.12%). PD‐(L)1 inhibitors were taken as first‐line treatment in 433 patients (63.68%). Patients concurrent with digestive diseases, cardiovascular diseases, and respiratory diseases accounted for 46.32%, 30.15%, and 21.62% of the total population, respectively. Of 680 participants, a great majority of patients (85.00%) had a CCI score ≥ 3.

**TABLE 1:** Demographic and clinical characteristics of 680 patients receiving immunotherapy.

Clinical characteristics		*N* (%)
Age (years)	< 65	410 (60.29)
	≥ 65	270 (39.71)
Sex	Male	521 (76.62)
	Female	159 (23.38)
BMI (kg/m^2^)	< 24	510 (75.00)
	≥ 24	170 (25.00)
Smoking status	Ever/current smokers	279 (41.03)
	Never smokers	401 (58.97)
ECOG PS	0/1	623 (91.62)
	2/3	57 (8.38)
Tumor type	Head and neck neoplasms^a^	18 (2.65)
	Esophageal cancer	83 (12.21)
	NSCLC	180 (26.47)
	Gastric cancer	78 (11.47)
	Liver cancer	139 (20.44)
	Colorectal cancer	47 (6.91)
	Genitourinary cancer^b^	37 (5.44)
	Melanoma	17 (2.50)
	Hematologic cancer	7 (1.03)
	Pancreatic cancer/Biliary tract carcinoma	29 (4.26)
	Others^c^	54 (7.94)
Clinical stage	I/II	49 (7.21)
	III/IV	631 (92.79)
Distant metastasis	Yes	424 (62.35)
	No	256 (37.65)
ICI drugs	Anti‐PD‐1 drugs	658 (96.76)
	Anti‐PD‐L1 drugs	22 (3.24)
Local therapy^d^	Yes	66 (9.71)
	No	614 (90.29)
Line of immunotherapy	First‐line	433 (63.68)
	Second‐line	121 (18.24)
	Third‐line and beyond	85 (12.50)
	Neoadjuvant/adjuvant therapy	41 (6.03)
Previous treatment	Chemotherapy	136 (20.00)
	Targeted therapy	46 (6.76)
	Surgery	229 (33.68)
	Radiotherapy	78 (11.47)
	Interventional therapy	91 (13.38)
Treatment type	Immunotherapy	278 (40.88)
	Immunotherapy + chemotherapy	285 (41.91)
	Immunotherapy + targeted therapy	143 (21.03)
Comorbidities	Respiratory diseases^e^	147 (21.62)
	Cardiovascular diseases^f^	205 (30.15)
	Nephropathy^g^	96 (14.12)
	Endocrine diseases^h^	118 (17.35)
	Digestive diseases^i^	315 (46.32)
	Hepatitis B/hepatitis B surface antigen carriers	144 (21.18)
	Others^j^	94 (13.82)
CCI score	≥ 3	578 (85.00)
	< 3	102 (15.00)
IrAEs	Yes	330 (48.53)
	No	350 (51.47)

Abbreviations: Anti‐PD‐1, anti‐programmed death 1; anti‐PD‐L1, anti‐programmed death ligand 1; BMI, body mass index; CCI, the Charlson comorbidity index; ECOG PS, Eastern Cooperative Oncology Group Performance Status; ICI, immune checkpoint inhibitor; irAEs, immune‐related adverse events; NSCLC, non‐small cell lung cancer.

^a^ Head and neck neoplasms included nasopharyngeal carcinoma, thyroid cancer, submandibular cancer, laryngeal cancer, tongue cancer, and lip cancer.

^b^ Genitourinary cancer included kidney cancer, bladder cancer, ureteral cancer, cervical cancer, and endometrioid adenocarcinoma.

^c^ Others included breast cancer, small cell lung cancer, thymic cancer, pulmonary mucoepidermoid carcinoma, and duodenal cancer.

^d^ Local therapy included radiotherapy and interventional treatment.

^e^ Respiratory diseases included chronic obstructive pulmonary disease, pulmonary hypertension, pneumonia, emphysema, tuberculosis, pleural effusion, asthma, and bronchitis.

^f^ Cardiovascular diseases included hypertension, coronary heart disease, cardiac insufficiency, and arrhythmia.

^g^ Nephropathy included renal insufficiency, renal cyst, and hydronephrosis.

^h^ Endocrine diseases included diabetes, Hashimoto's thyroiditis, hyperuricemia, hyperlipidemia, and gout.

^i^ Digestive diseases included hepatic insufficiency, cirrhosis, portal hypertension, cholecystitis, intestinal obstruction, gastritis, and ascites.

^j^ Others included syphilis, cerebral infarction, epilepsy, hypoproteinaemia, myelosuppression, electrolyte disorders, and depression.

### 3.2 Summary of irAEs

**FIGURE 2:**
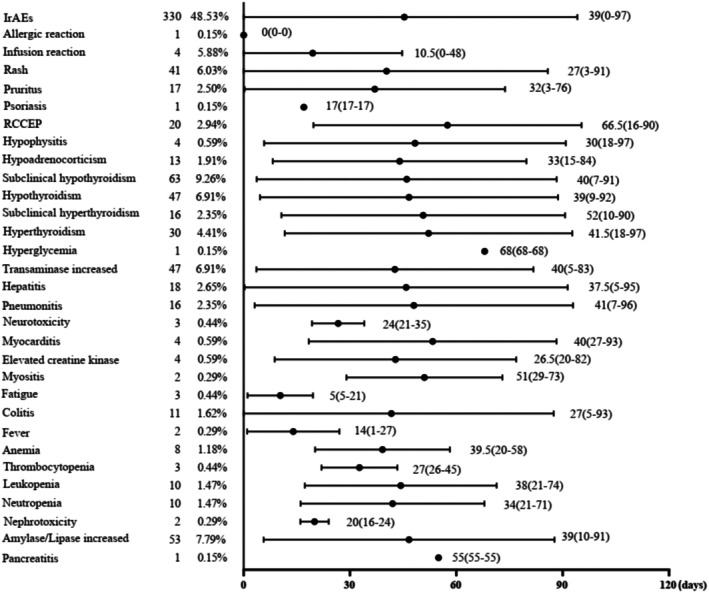
Time to onset of irAEs stratified by systems/organs. IrAEs, immune‐related adverse events; RCCEP, reactive cutaneous capillary endothelial proliferation.

In 330 (48.53%) patients, 455 different types of irAEs were reported, of which 52 events were grade 3 or higher in severity. The incidence rate of severe irAEs was 6.32% (43/680). There were no fatalities attributed to irAEs. Among patients with irAEs, 256 patients (37.65%) developed a single irAE, whereas 74 (10.88%) suffered from multisystem irAEs. For organ‐related toxicities, endocrinal toxicities exhibited the highest incidence rate (174/680, 25.59%), closely followed by dermatological (79/680, 11.62%) and liver (65/680, 9.56%) toxicities. Among all grades of irAEs, subclinical hypothyroidism (63/680, 9.26%), increased amylase/lipase (53/680, 7.79%), hypothyroidism (47/680, 6.91%), increased transaminase (47/680, 6.91%), and rash (41/680, 6.03%) were the most prevalent. The most common severe irAEs (grade ≥ 3) were rash (12/680, 1.76%), hepatitis (10/680, 1.47%), pneumonitis (6/680, 0.88%), and colitis (5/680, 0.74%). The median time to any irAE onset was 39 (range 0–97) days. Figure 2 shows the time to onset of each irAE. IrAEs caused treatment interruption in 49 (7.21%) patients, and discontinuation was permanent in 22 (3.24%) of them. There were 64 patients treated with systemic steroids (9.41%).

### 3.3 Development of Prediction Model for Risk Assessment of irAEs Based on LASSO‐Logistic Regression Analysis

**FIGURE 3:**
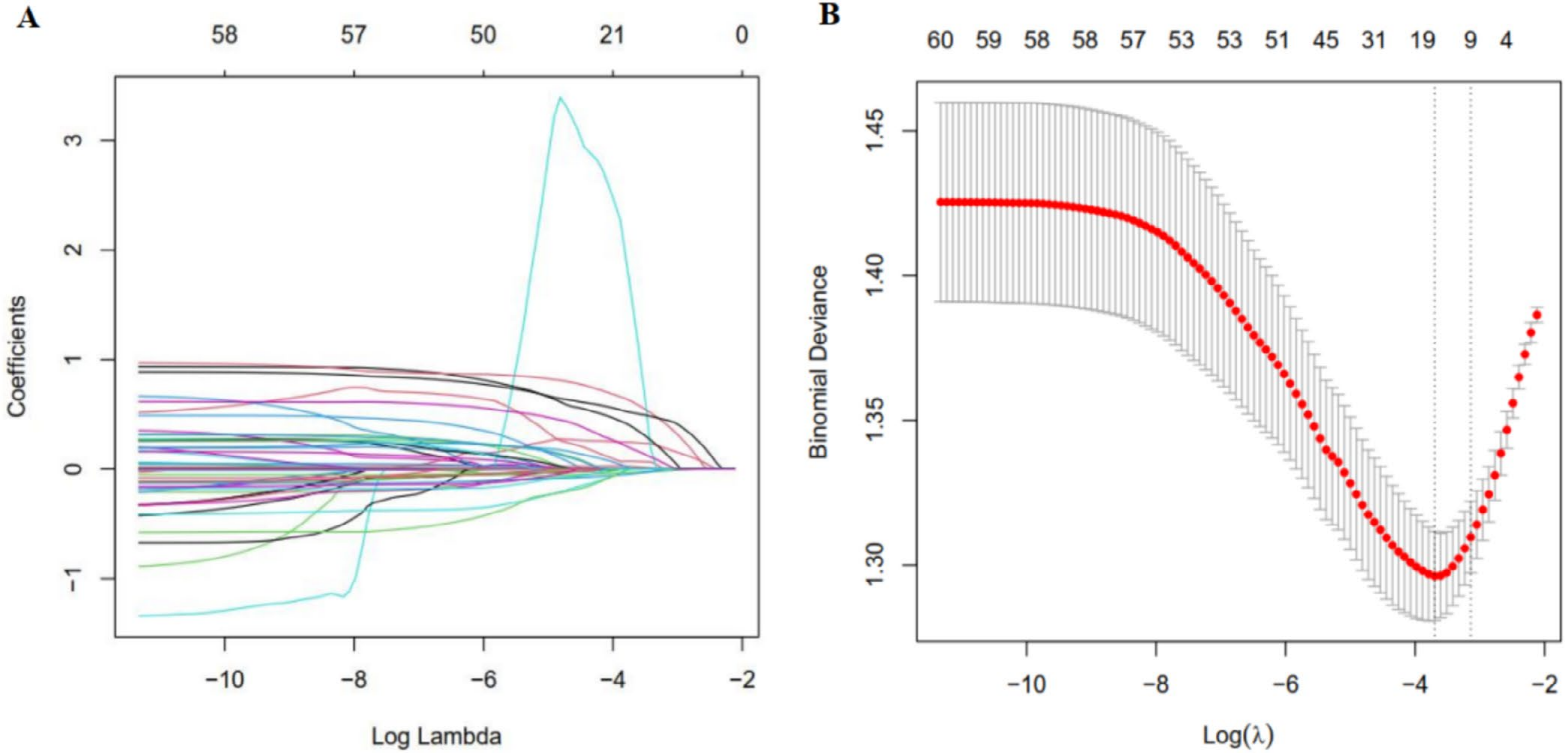
Variables selection using the LASSO regression analysis with 10‐fold cross‐validation. (A) The variation characteristics of the coefficient of variables; (B) The parameter λ selection of deviance in the LASSO regression based on the minimum criteria (left dotted line) and the 1‐SE criteria (right dotted line). LASSO, logistic least absolute shrinkage and selection operator; SE, standard error.

**FIGURE 4:**
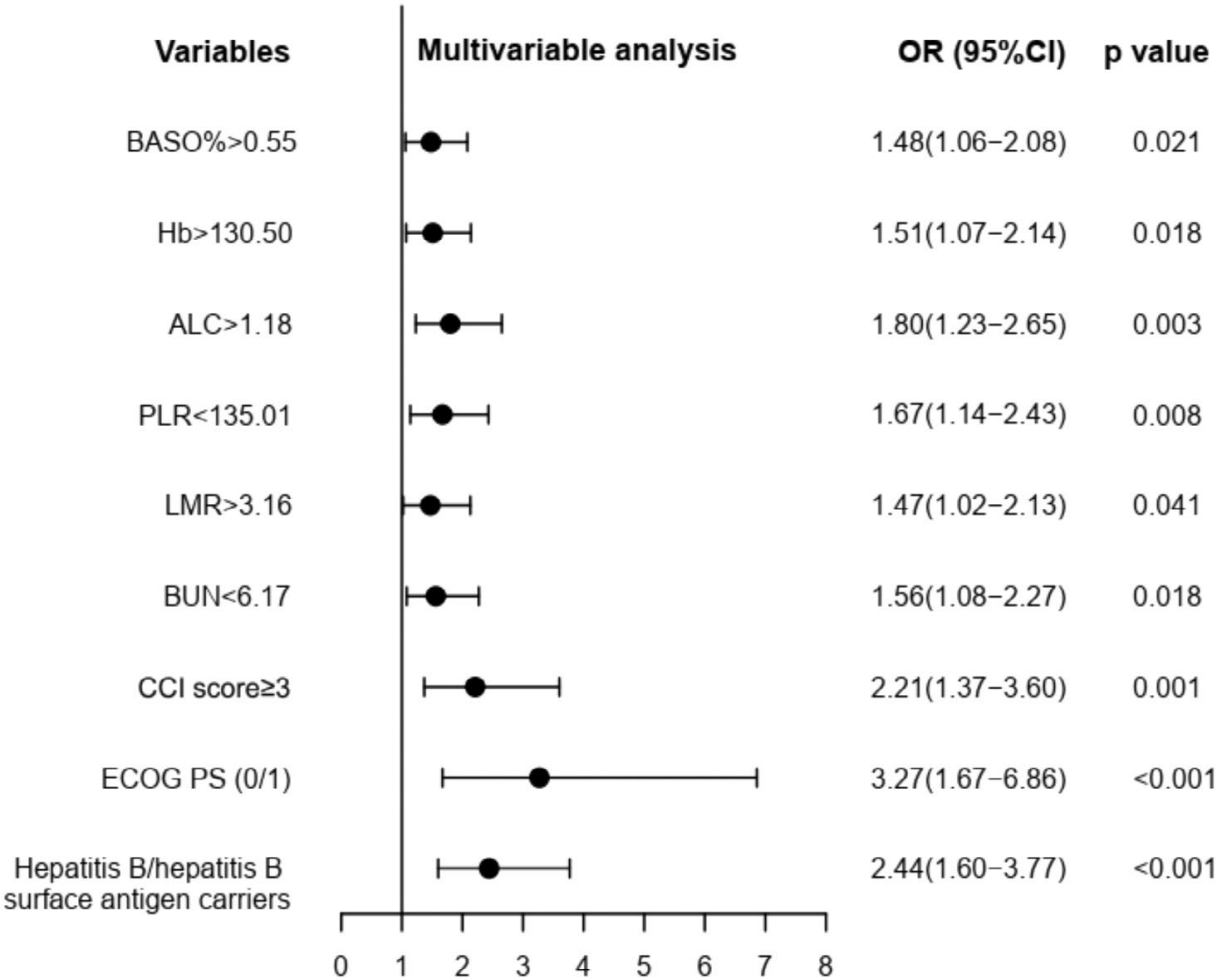
Multivariable logistic regression analysis of risk factors for occurrence of irAEs. ALC, absolute lymphocyte count; BASO%, basophil percentage; BUN, blood urea nitrogen; ECOG PS, Eastern Cooperative Oncology Group Performance Status.; Hb, hemoglobin; LMR, lymphocyte‐to‐monocyte ratio; PLR, platelet‐to‐lymphocyte ratio.

LASSO regression analysis was used to filter 61 parameters including demographic characteristics, clinical characteristics, laboratory findings and the variation characteristics of the coefficient of these variables were shown in Figure 3A. We utilized the 10‐fold cross‐validation method to select the penalty term, lambda(*λ*). When log(*λ*) was −1.606 (*λ* = 0.02475028), the error of the model was minimized and 14 variables were selected (Figure 3B). The screened variables included basophil percentage (BASO%), hemoglobin (Hb), absolute lymphocyte count (ALC), absolute basophil count (ABC), lymphocyte percentage (LYM%), NLR, PLR, PNI, LMR, blood urea nitrogen (BUN), CCI score, ECOG PS, hepatitis B/hepatitis B surface antigen carriers and liver cancer (Table 2). The logistic regression analysis with stepwise backward methods was further adopted to build a model based on variables screened by LASSO regression. At last, BASO% > 0.55, Hb > 130.50, ALC > 1.18, PLR < 135.01, LMR > 3.16, BUN < 6.17, CCI score ≥ 3, ECOG PS 0/1, and hepatitis B/hepatitis B surface antigen carriers were included to build the prediction model (Figure 4).

**TABLE 2:** The comparison of variables selected by LASSO regression analysis between irAEs and non‐irAEs groups.

Variables	IrAEs group	Non‐irAEs group	*p*
BASO% > 0.55	161 (48.79)	129 (36.00)	0.002
Hb > 130.50	162 (49.09)	109 (31.14)	< 0.001
ALC > 1.18	250 (75.76)	197 (56.29)	< 0.001
ABC > 0.025	228 (69.10)	205 (58.57)	0.004
LYM% > 20.85	214 (64.85)	161 (46.00)	< 0.001
NLR < 3.36	219 (66.36)	170 (48.57)	< 0.001
PLR < 135.01	158 (47.88)	91 (26.00)	< 0.001
PNI > 44.98	211 (63.94)	165 (47.14)	< 0.001
LMR > 3.16	185 (56.06)	135 (38.57)	< 0.001
BUN < 6.17	251 (76.06)	238 (68.00)	0.019
CCI score ≥ 3	293 (88.79)	285 (81.43)	0.007
ECOG PS 0/1	318 (96.36)	305 (87.14)	< 0.001
Hepatitis B/hepatitis B surface antigen carriers	98 (29.70)	46 (13.14)	< 0.001
Liver cancer	90 (27.27)	49 (14.00)	< 0.001

Abbreviations: ABC, absolute basophil count; ALC, absolute lymphocyte count; BASO%, basophil percentage; BUN, blood urea nitrogen level; CCI, the Charlson comorbidity index; ECOG PS, Eastern Cooperative Oncology Group Performance Status; Hb, hemoglobin; LMR, lymphocyte‐to‐monocyte ratio; LYM%, lymphocyte percentage; NLR, neutrophil‐to‐lymphocyte ratio; PLR, platelet‐to‐lymphocyte ratio; PNI, prognostic nutrition index.

### 3.4 Visualization and Evaluation of the Prediction Model

**FIGURE 5:**
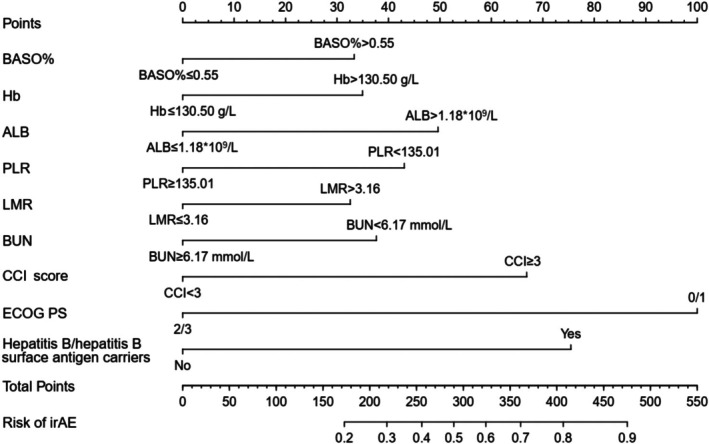
Nomogram construction for predicting irAEs based on the high‐risk variables. ALC, absolute lymphocyte count; BASO%, basophil percentage; BUN, blood urea nitrogen; ECOG PS, Eastern Cooperative Oncology Group Performance Status; Hb, hemoglobin; LMR, lymphocyte‐to‐monocyte ratio; PLR, platelet‐to‐lymphocyte ratio.

**FIGURE 6:**
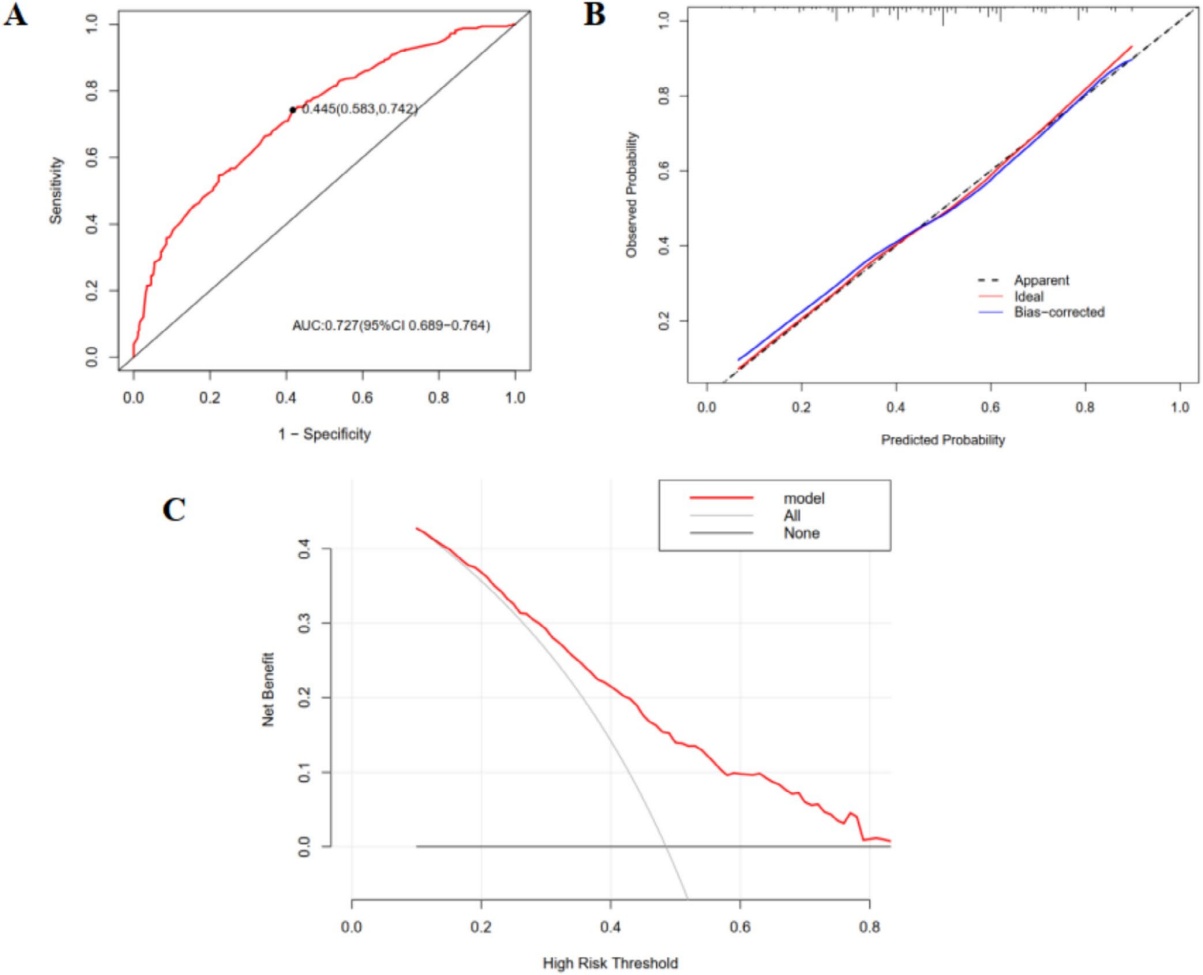
Evaluation of the prediction model for incidence of irAEs. (A) ROC curve for the prediction model. (B) calibration curve for the prediction model. (C) DCA for the prediction model. DCA, decision curve analysis; ROC, receiver operating characteristic.

**FIGURE 7:**
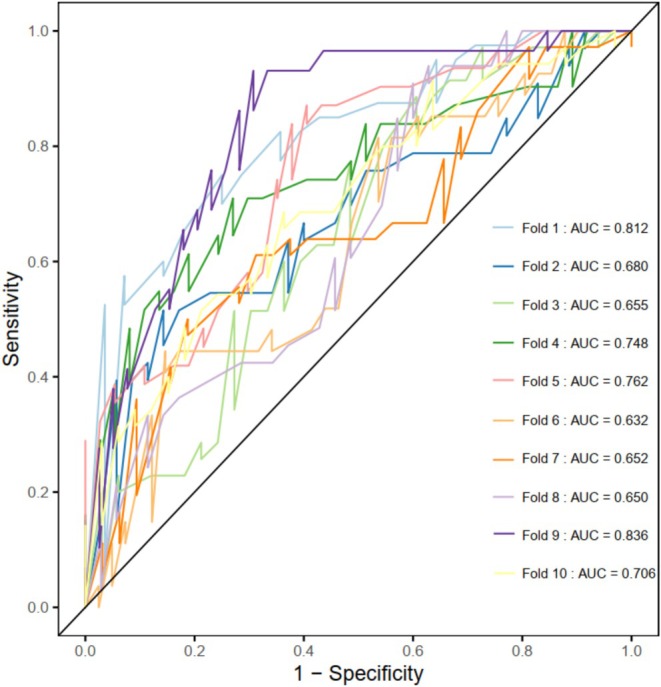
ROC curve for the 10‐fold cross‐validation of prediction model. AUC, area under the curve; ROC, the receiver operating characteristic.

As shown in Figure 5, we developed a nomogram to visualize the established prediction model. In the nomogram, each predictor has corresponding “points,” then the probability can be obtained by the total score through adding the points of nine predictors. The longer line length, the greater contribution of risk factors to irAEs. The *C* index of the model was 0.727 (95% confidence interval, 0.689–0.764), which indicates moderate accuracy of the nomogram in predicting probability of irAEs. It was also demonstrated by the receiver operating characteristic (ROC) curve (Figure 6A). The calibration curve demonstrated the probability of irAE predicted by our model was positively correlated with actual probability of irAE. And our model had a good degree of calibration (Figure 6B). Besides, we applied decision curve analysis (DCA) to explore the model's potential clinical benefit and results showed that it displayed significant net benefits when the threshold probability was between 15% and 85% (Figure 6C). Additionally, 10‐fold cross‐validation for internal validation was used to evaluate the robustness of the predictive model, and the area under the curve (AUC) was 0.713 (Figure 7).

## 4 Discussion

ICIs have emerged as a milestone of cancer therapy, but unfortunately, irAEs are inevitably the barriers to efficacies. Our study accurately depicted the real‐world incidence and types of irAEs after ICIs administration in pan‐cancer patients. In this study, we investigated 680 cancer patients who received anti‐PD‐1/PD‐L1 immunotherapy and found a 48.53% incidence of irAEs, including 6.32% grade 3–5 irAEs. It was similar to existing observational studies, which had reported an incidence of 29.3%–49.4% for all grades and 2.0%–7.1% for grades 3–5 in patients receiving ICIs [23, 24]. Consistent with previous findings, endocrinal toxicities, particularly thyroid dysfunctions, were the most common irAEs and accounted for 34.29% of all irAEs in our study [25, 26].

Our primary analysis focused on the first 90 days after the initiation of ICIs, as studies have reported the highest incidence of irAEs during this period [21, 22]. The occurrence of early irAEs may significantly compromise patient adherence to immunotherapy regimens by undermining treatment confidence. Therefore, our research is committed to identifying high‐risk patients who may develop irAEs within 3 months. Additionally, the identification of late‐onset events warrants particular attention. Future studies should incorporate extended follow‐up windows (≥ 6 months).

Unlike other studies, we applied LASSO regression analysis to screen meaningful factors for the occurrence of irAEs. This method, which reduces regression coefficients to identify potential variables, is superior to selecting indicators based on the strength of the univariable association with the outcome. Furthermore, our study established a prediction model based on nine variables and the AUC value was 0.727, with a cut‐off value of 0.445.

Extant literature reveals that several factors have been correlated with the incidence of irAEs across various studies. Circulating blood cell counts and ratios, such as ALC, absolute monocyte count (AMC), platelet count, NLR, PLR and LMR, have received widespread attention due to wide availability, low cost, and easy interpretation. Patients with high baseline ALC (ALC > 1.18 × 10^9^/L) had a greater risk for irAEs compared to those with low baseline ALC. A positive correlation between high ALC at baseline and ICIs‐related toxicities has been discovered by several researchers, such as Xu, Teijeira et al. [27, 28]. Several other studies have identified that lower levels of PLR at baseline were associated with higher rate of irAEs [29, 30]. In a study consisting of 470 patients with various types of malignancies, Michailidou et al. [31] demonstrated that patients with AMC > 0.29 × 10^9^/L and LMR > 1.37 at baseline had an increased risk of irAEs. Consistent with their results, patients with high level of PLR and low level of LMR in our study also had fewer opportunities to experience irAEs. It may suggest that peripheral lymphocytosis can lead to an imbalance of T cell subsets, including changes in CD8^+^ T cells and CD4^+^ T cells, which may result in hyperinflammation and autoimmunity, thereby increasing the risk of toxicities [32, 33].

Quite a lot of investigations suggested that baseline absolute eosinophil count (AEC) was a strong predictor of irAEs in patients undergoing treatments with PD‐(L)1 inhibitors [34, 35]. However, our results indicated there was no association between AEC and the development of irAEs, but patients with a higher BASO% were prone to developing irAEs. Besides, several studies revealed high basophil counts seem to be favorable for the outcome [36, 37]. But there were no studies to illustrate the connection between basophil counts and the occurrence of irAEs. The relationship between basophils and irAEs requires further investigation. Surprisingly, we found that BUN level at baseline was also identified as a significant risk factor, a novel finding that has never been reported before. Considering the BUN level can reflect the level of kidney function, we speculated that renal insufficiency could potentially result in dysregulation of immune homeostasis, ultimately leading to irAEs. To the best of our knowledge, the association between BASO% and BUN levels with irAEs has not been previously reported in the literature. This gap may arise from the single‐center nature of our study, which could introduce selection bias and limit the generalizability of these findings. Elucidating the underlying biological mechanisms linking BASO% and BUN to irAEs pathogenesis is critical for validating their potential as predictive biomarkers. While our retrospective analysis only identified a statistical correlation, we will carry out prospective, multi‐center studies to ascertain whether these factors are indeed significant risk factors for the occurrence of irAEs in the future.

Salman et al. [38] discovered that lower Hb level was a significant predictor of irAEs occurrence. However, unlike their study, our research revealed that a higher Hb level (Hb > 130.5 g/L) was associated with an increased probability of experiencing irAEs. How this affects the toxicities of ICIs is still unclear. CCI, which serves as a comprehensive index for assessing multiple morbidities, is a good indicator of a patient's global status and has been investigated as a potential predictive factor for development of irAEs [39]. Onur et al. [40] revealed that there was a significant relationship between higher CCI scores and irAEs in lung cancer and renal cell cancer patients. Similarly, our research indicated that irAEs were more likely to occur in patients with CCI score ≥ 3, regardless of tumor type. Patients' fragilities may rise as comorbidities increase resulting in sensitivities to toxicities.

Notably, we found that ECOG PS ≥ 2 was significantly related to a lower incidence of irAEs of any grade, which is similar to previous reports [35, 41, 42]. Physical status has been widely recognized as prerequisites for antitumor responses. If irAEs arise from the pharmacodynamic activity of ICIs, a poor PS could suggest a state of repressed immune reactivity leading to a lower likelihood of developing irAEs [41].

In the majority of pivotal trials that led to initial approvals of ICIs, patients with prior infections with hepatitis B virus (HBV) were traditionally excluded [43]. The safety data of immunotherapy in this special population of HBV‐infected patients with cancers is quite limited in previous studies. Some disclosed that there was no difference in the incidence of ICIs‐induced toxicities between patients with HBV infection and those without [44, 45, 46]. Compared with patients without HBV infection, those infected had a higher risk of irAEs in our study. ICIs may potentially trigger the reactivation of HBV, leading to the massive release of cytokines and chemokines—the essential components of the immune system. These inflammatory factors recruit regulatory immune cells, which may exacerbate immune dysregulation and contribute to the development of irAEs [47, 48]. Besides, there is also a study indicating that patients with baseline HBV‐DNA > 500 IU/mL are more prone to irAEs [49]. Our conclusion that the occurrence of irAEs was associated with HBV infection may suggest that cancer patients with HBV infection should be monitored closely when receiving ICIs.

Building upon the identified risk factors associated with the onset of irAEs, several researchers have endeavored to construct prediction models that integrate these variables. There have been a few prediction models for the occurrence of irAEs in cancer patients receiving ICIs. In 2022, Xu et al. [28] built a model based on treatment lines, aspartate aminotransferase, lactate dehydrogenase, ALC, and SII. Gao et al. [50] established a clinical prediction model incorporating treatment lines, combination therapy of ICIs, ECOG PS, NLR, platelet, and lymphocyte. However, both models were limited to NSCLC patients and cannot be effectively generalized to other populations. Zhao et al. [51] constructed a clinical prediction model based on the duration of treatment, hepatic metastases, IL2 levels, and IL8 levels to estimate risks of irAEs. Recently, Yu et al. had utilized plasma lipidomics to develop a rapid and effective prediction model for identifying irAEs, and its indicators mainly included lysophosphatidylcholines (LPC)‐18:2, phosphatidylcholines (PC)‐40:6, LPC‐22:6, LPC‐O‐18:0, phosphatidylserine (PS)‐38:0, PC‐38:6, PC‐37:6, PC‐36:5, LPC‐17:0. This biomarker panel exhibited an excellent AUC of 0.940 in the validation cohort [15]. Lim et al. identified that 11 cytokines were significantly elevated in patients with severe immune toxicity at baseline and early during treatment. They developed a cytokine toxicity score using these 11 cytokines, which achieved an AUC of 0.78 and demonstrated good predictive performance [14]. However, these models' applicability was constraining because cytokine levels or lipidomic profiles were infrequently measurable in clinical settings. Compared to the above models, although the performance of our model is indeed inferior to theirs, we used clinical and hematological parameters that are more easily available in real practice. Besides, we enrolled a large number of pan‐cancer patients in the study; thus, our model can be anticipated to be applied generally. In addition, we conducted prospective experiments to explore the relationship between specific cytokines (e.g., IL‐21, SCF) and irAEs, but no significant associations have been identified to date. We will continue to collect samples to further investigate this correlation.

The tumor microenvironment (TME) plays a crucial role in tumor progression and prognosis [52, 53, 54]. While some studies establish critical links between TME factors (e.g., hypoxia, PLIN3, FNDC4) and oncogenesis [55], their specific association with irAEs remains unexplored. We plan to investigate how TME dynamics may influence irAEs pathogenesis in future studies, with the aim of elucidating potential mechanistic connections.

Our study exclusively employs linear learning algorithms to develop predictive model. In contrast, prior research has demonstrated non‐linear machine learning models have indeed demonstrated impressive performance in various biomedical prediction tasks, including in oncology, as evidenced by work such as Ogunleye et al. and Seyednasrollah et al. [56, 57]. Even, fully automated machine learning for predictive modeling is becoming a reality, such as Just Add Data Bio (JADBio) [58]. We will explore non‐linear algorithms (e.g., XGBoost, neural networks) in future work using expanded datasets to assess potential performance gains while maintaining interpretability where feasible.

However, there are several limitations in our study. Firstly, due to it being a single‐center analysis and lack of external validation, our findings may not be applicable in some other circumstances. Secondly, we only analyzed irAEs within 3 months after treatment initiation. But irAEs may be discovered later than 3 months; incidence calculated in our investigation was definitely lower than actual irAEs incidence. Thirdly, we did not include PD‐L1 expression levels and tumor mutational burden in the analysis accounting for missing data in many patients. Undoubtedly, further multi‐center investigations about irAEs prediction models are urgent to be conducted and validated. Last but not least, the use of non‐nested cross‐validation for hyperparameter tuning and performance evaluation may introduce optimistic bias in the reported AUC. Future studies should employ nested cross‐validation or external validation cohorts for stricter assessment.

## 5 Conclusion

In this study, we established a risk assessment model for irAEs in cancer patients undergoing anti‐PD‐(L)1 therapy. The nomogram model incorporating indicators available clinically can be applied as a useful aid for clinicians to predict the risk of irAEs. It can facilitate more effective monitoring of the focused risk population and ultimately improve prognosis. We look forward to expanding the sample size and conducting multi‐center prospective studies to optimize the model in the future.

## Author Contributions

**Panpan Jiao:** conceptualization, methodology, data curation, formal analysis, writing – original draft, writing – review and editing. **Lijuan Xue:** data curation. **Weijuan Tan:** data curation. **Quan Chen:** writing – review and editing. **Shan Lin:** writing – review and editing. **Min Song:** writing – review and editing, supervision. **Chunling Ma:** writing – review and editing. **Juan Zhan:** conceptualization, methodology, writing – original draft, writing – review and editing, funding acquisition, supervision.

## Funding

This work was supported by Xiamen Medical and Health Science and Technology Project (grant number 3502Z20244ZD1062).

## Ethics Statement

This study was approved by the Medical Ethics Board of Zhongshan Hospital affiliated to Xiamen University on July 7, 2023 in Xiamen, China (ID: xmzsyyky 2023‐129). Special approval has been obtained from the Institutional Review Board for this retrospective study, and obtaining patients' informed consent is not required.

## Conflicts of Interest

The authors declare no conflicts of interest.

## Data Availability

The data used and/or analyzed during the current study are available from the corresponding author on reasonable request.
